# Report on the 2nd MObility for Vesicle research in Europe (MOVE) symposium—2024

**DOI:** 10.1002/2211-5463.70112

**Published:** 2025-08-29

**Authors:** Dorival Mendes Rodrigues‐Junior, Michaela Kocholata, Marilena E. Lekka, Olga Janouskova, Moran Yadid, Maja Kosanovic

**Affiliations:** ^1^ Department of Medical Biochemistry and Microbiology, Science for Life Laboratory, Biomedical Center Uppsala University Sweden; ^2^ Centre of Nanomaterials and Biotechnologies, Faculty of Science Jan Evangelista Purkyne University in Usti nad Labem Czech Republic; ^3^ Chemistry Department University of Ioannina Greece; ^4^ The Azrieli Faculty of Medicine Bar Ilan University Safed Israel; ^5^ Institute of Nanotechnology and Advanced Materials Bar‐Ilan University Ramat Gan Israel; ^6^ Institute for the Application of Nuclear Energy INEP University of Belgrade Republic of Serbia

**Keywords:** biomarkers, exosomes, extracellular vesicles, intercellular communication, microvesicles, research mobility

## Abstract

The 2nd MObility for Vesicle research in Europe (MOVE) Symposium, held in Belgrade—Serbia, from October 8 to 11, 2024, showcased the dynamic and interdisciplinary nature of extracellular vesicles (EVs) research in Europe. Organized by eight National EV Societies under the MOVE initiative, the event gathered over 280 attendees from 28 countries, promoting collaboration and scientific exchange. The symposium featured eight keynote lectures, 48 oral and 126 poster presentations, and sessions dedicated to EV‐related tools and industry innovations. The scientific program was structured around seven core themes: EV biogenesis and signal transmission, roles of EVs in health and disease, EV‐based biomarkers, interspecies communication, novel EV preparation and analysis techniques, therapeutic and regenerative applications, and the manufacturing of native and engineered EV products. Supported by 18 sponsors and the Ministry of science, technological development and innovation of the Republic of Serbia, the symposium also highlighted the MOVE Fellowship Program and offered rich networking opportunities. This landmark event reinforced MOVE's promising mission to promote excellence, mobility, and resource sharing in EV research across Europe.

## Graphical abstract

The 2nd MObility for Vesicle research in Europe (MOVE) Symposium in Belgrade brought over 280 attendees from 28 countries to advance extracellular vesicle (EV) research. Featuring keynotes, presentations, and industry sessions, it covered EV biogenesis, biomarkers, therapies, and manufacturing. The event promoted collaboration, showcased innovations, and reinforced MOVE's mission to promote excellence and mobility in EV science across Europe.

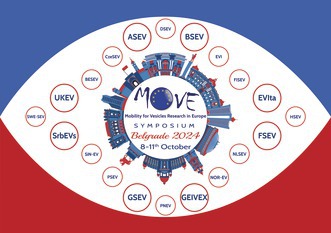

**Abbreviations**
3D: three‐dimensional
ALS: amyotrophic lateral sclerosis
cGRP: gamma‐carboxylated Gla‐rich protein
CM‐EV: cardiomyocyte‐derived extracellular vesicle
CRC: colorectal cancer
EMT: epithelial‐mesenchymal transition
EMV: erythrocyte membrane‐based vesicle
EV: extracellular vesicle
Ex‐EV: exercise‐induced extracellular vesicle
HC: hypercholesterolemia
HDP: how host defense peptide
MDR: multidrug‐resistant
miRNA/miR: microRNA
MOVE: MObility for Vesicle research in Europe
MSC‐EV: mesenchymal stem cell‐derived extracellular vesicle
NanoEs: nanoerythrosomes
NEVs: European National EV Societies
NTA: nanoparticle tracking analysis
PDEV: plant‐derived extracellular vesicle
PL‐EV: platelet‐derived extracellular vesicle
PTX: paclitaxel
TGF‐β: transforming growth factor‐β
TNBC: triple‐negative breast cancer
uEV: urinary extracellular vesicle
WGSM: waveguide scattering microscopy

The research field of extracellular vesicles (EVs) has experienced a remarkable surge of scientific interest due to growing evidence of EVs' pivotal role in intercellular communication and their exceptional potential as diagnostic and therapeutic tools. The EV field is evolving into an interdisciplinary frontier of biomedical research, and its rapid growth underscores the need for platforms that support scientific exchange, methodological harmonization, and cross‐disciplinary collaboration. Hence, numerous national and international EV societies have been established, and the MObility for Vesicle research in Europe (MOVE) was launched as an informal consortium of European National EV Societies (NEVSs). MOVE's goal is to foster communication, mobility of young researchers, and joint activities across Europe by promoting scientific excellence, resource sharing, and training opportunities for EV researchers through collective action.

To expand these objectives, MOVE's 1st Symposium was held in 2023 in Málaga, Spain, organized by four NEVSs: GEIVEX (Spain), EVIta (Italy), GSEV (Germany) and UKEV (United Kingdom) [1]. Building on this success, the 2nd MOVE Symposium took place in Belgrade, Serbia, from 8 to 11 October 2024. At this time, the meeting was organized by eight NEVSs: SrbEVs (Serbia) as the local organizer, alongside ASEV (Austria), BSEV (Baltic countries), FSEV (France), EVIta, GEIVEX, GSEV, and UKEV (Fig. 1). The program featured eight keynote lectures from leading researchers in the EV field, seven topics were distributed among 48 oral and 126 poster presentations, two sessions dedicated to EV‐related tools with 10 industry presentations, and two inspiring round tables. The 2nd MOVE Symposium welcomed over 280 attendees from 28 countries across Europe but also from Asia and the Americas, including MOVE Fellowship presenters (Fig. 2). Notably, this meeting was supported by 18 sponsors, all prominent companies in the EV field, and the Ministry for Science, Technological Development and Innovation of the Republic of Serbia. A highlight was the networking evening at Gardoš restaurant, near the historic Millennium Tower. The 2nd symposium received excellent feedback, reinforcing MOVE's commitment to supporting mobility and collaboration, especially for early‐career EV researchers.

**Fig. 1:**
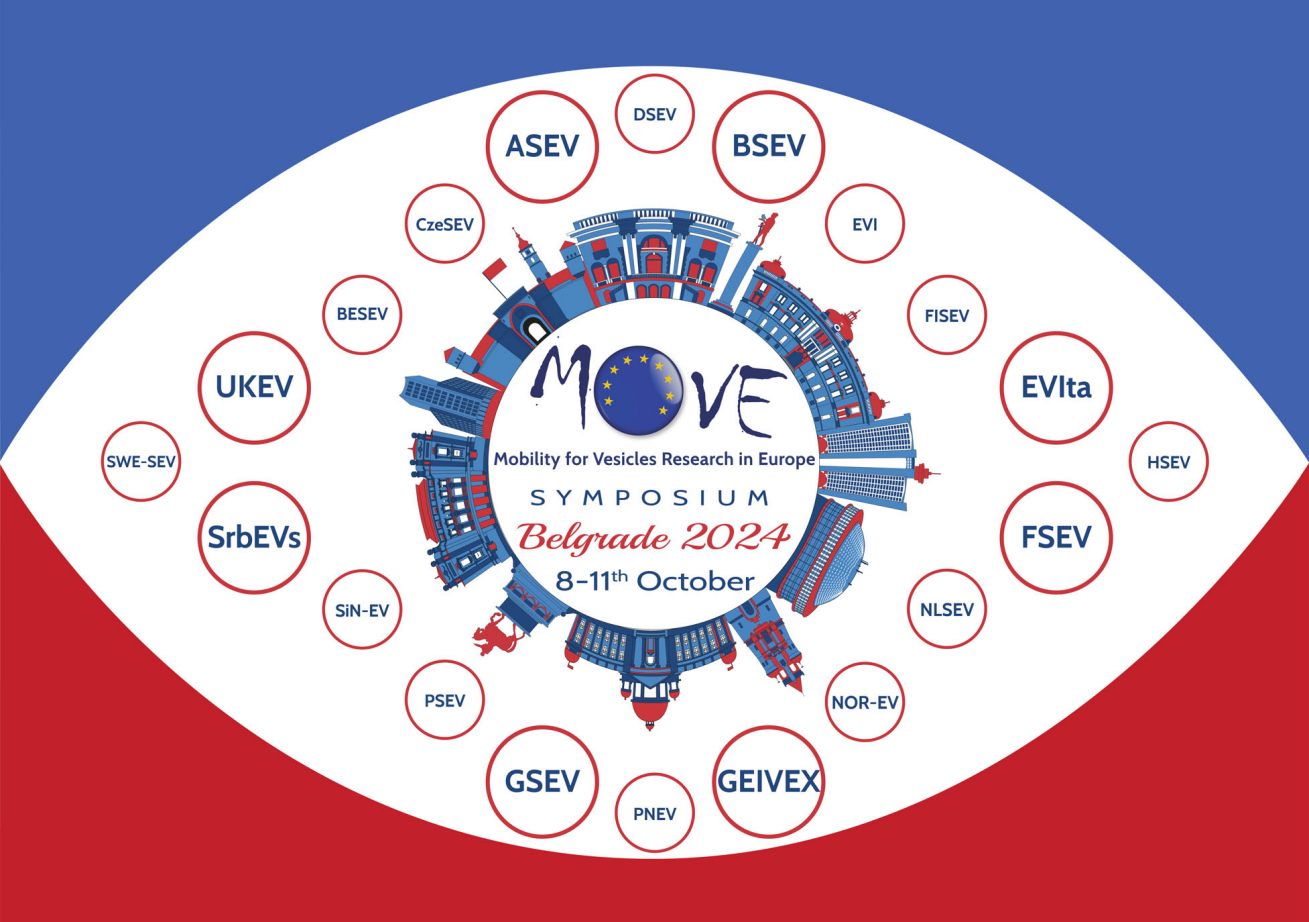
The 2nd MOVE Symposium logo. The colors were inspired by the city of Belgrade's coat of arms, while the center circle depicts prominent architectural and historic landmarks of Belgrade. The bigger circles represent co‐organizing societies and the smaller ones all other MOVE societies. The logo can be interpreted as an insight (“eye” shape) into the communication of the cell (the city of Belgrade as the place of gathering of societies, with buildings representing heterogenous molecules on cell's surface) and the extracellular vesicles (circles with NEVSs’ logos). Design by Maja Kosanović.

**Fig. 2:**
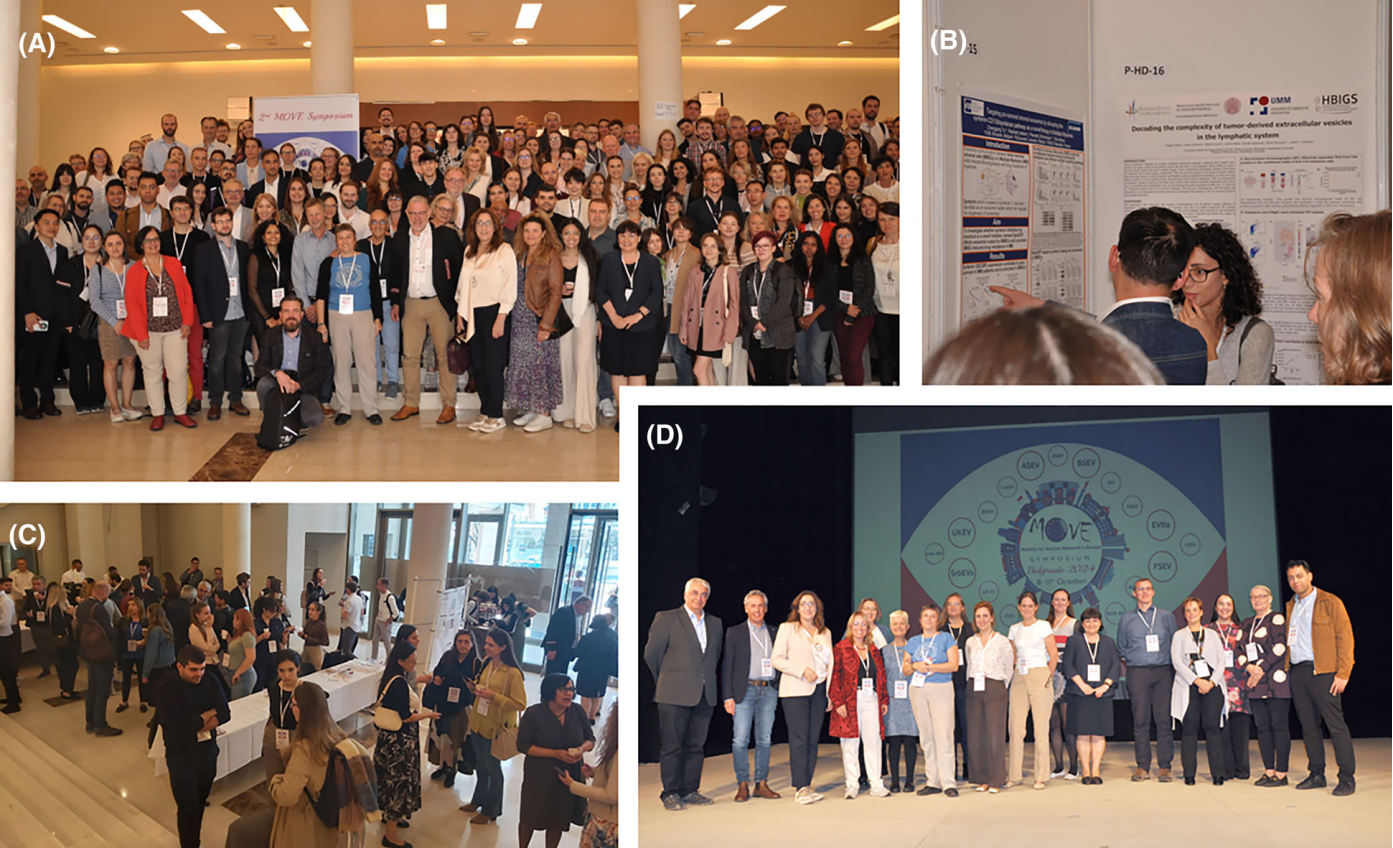
Brief overview of the 2nd MOVE Symposium. (A) Over 280 participants attended the 2nd MOVE Symposium held in Belgrade—Serbia, from October 8 to 11, 2024. (B) Participants interacting during the poster session. (C) Participants promoting network during coffee break. (D) Members of the European National EV Societies (NEVSs).

## Keynote speakers

### Frederik J. Verweij

Verweij presented innovative molecular tools to study how multivesicular bodies (MVBs) mature and release exosomes. Using live‐cell imaging and pH‐sensitive reporters, he tracked individual MVBs and revealed a “step‐wise maturation” process. MVBs pause in a peri‐nuclear “decision zone” before either fusing with the plasma membrane to release exosomes or being degraded in lysosomes. A Rab GTPase relay (Rab7a → Arl8b → Rab27a) and ER contact sites guide MVBs toward secretion, while RNF26 and Rab2a regulate the balance between release and degradation [2]. Cargo can be loaded into MVBs via both endocytic recycling and direct ER/Golgi routes, with novel tetraspanins (TSPAN2/3) supporting this dual pathway [3, 4]. These findings offer a detailed framework for controlling EV secretion and identify new molecular targets for therapeutic intervention.

### Tobias Tertel

Tobias Tertel outlined a rigorous framework for using EVs in clinical diagnostics, emphasizing their potential as immune‐state biomarkers across various diseases. Drawing on data from over 3000 biofluid samples covering conditions like COVID‐19, stroke, cancer, and joint infections, he demonstrated that EVs, especially when analyzed via imaging flow cytometry, can reveal consistent immune signatures. Inflammatory diseases showed pro‐inflammatory EV profiles, while cancers exhibited immune‐modulatory traits, suggesting EVs reflect the body's immune tone rather than disease‐specific markers. Tertel showed that EV markers (CD24^+^, CD13^+^, CD82^+^) correlated with COVID‐19 disease severity, with CD24^+^ EVs indicating milder cases and potential for therapeutic use [5]. Additionally, in prosthetic joint infections, EVs (CD9^+^, CD82^+^) enabled rapid differentiation between septic and aseptic loosening, outperforming traditional cultures [6]. Tertel stressed that standardization in sample handling, antibody validation, and assay design is essential for reliable EV‐based diagnostics. His team's screening of over 600 antibodies, with fewer than 70 meeting criteria, highlighted the need for stringent protocols to unlock EVs' full clinical potential.

### D. Michiel Pegtel

Pegtel introduced circulating EVs from multiple myeloma patients as a promising, minimally invasive alternative to bone marrow biopsies. Central to his work is IsoSeek, a highly accurate small RNA sequencing method that minimizes technical bias and enables single‐nucleotide resolution detection of microRNAs (miRNAs) and their variants (isomiRs) from very small RNA samples [7]. Using IsoSeek, Pegtel's team analyzed plasma EVs from over 100 samples, distinguishing active disease from remission with high accuracy (AUC 0.98) and predicting treatment response to daratumumab (AUC 0.84). These predictive isomiRs were linked to key survival pathways involving MYC and BCL2, highlighting their biological and clinical relevance. Pegtel's study demonstrated that EV‐isomiRs reflect tumor molecular states, enabling non‐invasive, serial monitoring of patients and emphasized that combining IsoSeek with machine learning can generate powerful risk models to guide personalized treatment decisions, potentially transforming the clinical management of multiple myeloma and accelerating the development of personalized therapies.

### Kenneth W. Witwer

Witwer presented advancements in tracking EVs in rodents, primates, and humans. Using sensitive reporters like PalmBRET, EGFP–NanoLuc, and MemGlow dyes, his team achieved accurate EV detection and pharmacokinetic profiling in macaques, revealing longer plasma half‐lives and cerebrospinal fluid penetration within an hour [8, 9, 10]. Imaging showed EVs localizing in the liver and spleen after intravenous delivery, while intranasal administration resulted in minimal systemic uptake, highlighting EV challenges in crossing the blood–brain barrier. Flow cytometry revealed a conserved EV tropism toward B cells in both macaques and humans, suggesting that B cells act in EV delivery and clearance [11]. Witwer also addressed EV immunogenicity, as repeated high‐dose foreign EVs can trigger strong IgG responses, provoking the idea that EVs are immunologically “stealth”. Finally, he endorsed the MISEV 2023 guidelines [12], which call for stricter standards in EV research, especially for *in vivo* studies. His work provides essential tools and insights for advancing EV‐based therapeutics.

### Dhanu Gupta

Gupta presented how EVs are evolving from passive biomarkers into programmable delivery systems for precision therapies. His work addresses key challenges in EV manufacturing, cargo loading, and functional release by identifying novel EV‐sorting scaffold proteins, especially TSPAN2 and TSPAN3, which outperformed traditional markers like CD63. These scaffolds enable efficient, endogenous cargo loading without damaging physical methods [13]. By engineering producer cells and leveraging EV biogenesis pathways, Gupta's approach improves yield, uniformity, and compatibility with scalable production systems. Gupta also demonstrated successful delivery of CRISPR‐Cas9 ribonucleoproteins via EVs, achieving high gene‐editing efficiency without viral vectors [14]. To enhance intracellular release, his team developed the VEDIC platform, combining EV loading, pH‐sensitive cargo release, and membrane fusion capabilities [15]. This system showed strong therapeutic effects in mouse models, rivaling lipid nanoparticles but with biologically native components. Overall, Gupta's work positions EVs as a next‐generation therapeutic platform, offering high payload capacity, precise targeting, and safer delivery for complex biologics.

### Bernd Giebel

Giebel discussed the therapeutic potential of mesenchymal stem cell‐derived extracellular vesicles (MSC‐EVs), which offer immunomodulatory and regenerative effects across diseases like GvHD, stroke, COVID‐19, and sepsis [16]. MSC‐EVs are attractive as cell‐free therapies, but their clinical translation faces challenges due to functional variability among MSC and MSC‐EV preparations, even when derived from the same cell source. To address this, Giebel's team developed clonally expanded, immortalized MSC lines (ciMSCs) using a lentiviral hTERT‐based strategy. These ciMSCs produce EVs with consistent immunomodulatory activity and therapeutic effects *in vivo*, marking a key step toward standardized, GMP‐compliant production. However, further work is needed to refine upstream and downstream processing and establish robust functional testing to ensure clinical‐grade MSC‐EV products are safe, effective, and reproducible.

### Antonio Marcilla

Marcilla explored how EVs are a universal communication tool across biological kingdoms, focusing on parasite and plant‐derived EVs (PDEVs) [17, 18]. He highlighted how parasitic helminths, such as *Fasciola hepatica* and *Echinostoma caproni*, secrete EVs carrying proteins and miRNAs that modulate host immune responses, shifting the view of host–parasite interactions from passive to active molecular dialog. These EVs can suppress inflammation, reprogram immune cells, and even serve as vaccine platforms, offering protection in disease models like ulcerative colitis [19, 20, 21, 22].

Marcilla also introduced PDEVs from sources like grape and pomegranate, which show anti‐inflammatory and wound‐healing properties [22, 23]. Interestingly, PDEVs can be bioengineered to deliver drugs or RNA to parasites, acting as natural, non‐toxic nanocarriers [24]. He emphasized that EVs mediate communication also across kingdoms, such as plant–insect and insect–microbe interactions, opening new possibilities in agriculture and biotechnology. To fully harness these potentials, Marcilla called for standardized EV isolation, improved single‐vesicle analysis, and advanced imaging tools. His vision positions EVs as powerful tools for diagnostics, immunotherapy, and sustainable nanomedicine, bridging parasitology, immunology, and green technology.

### Rossella Crescitelli

Crescitelli presented groundbreaking work on EVs in solid tissues, using three‐dimensional (3D) electron tomography to capture *in situ* images of interstitial EVs in human liver metastases. This led to the development of a collagenase‐assisted isolation method that enables recovery of distinct EVs directly from frozen tissue samples, unlocking the potential of archived biobank material for retrospective studies [25, 26]. She demonstrated that tumor‐derived EVs can serve as cancer biomarkers, identifying a mitochondrial protein signature (MT‐CO2 and COX6c) detectable in melanoma, ovarian, and breast cancer patients [27]. Additionally, tumor EV‐DNA showed higher sensitivity for detecting cancer mutations than total circulating DNA, enhancing minimal residual disease monitoring [28].

Beyond diagnostics, Crescitelli introduced synthetic bacterial vesicles as safe, potent adjuvants. When combined with tumor EVs, they induced strong anti‐tumor immune responses and synergized with checkpoint inhibitors in mouse models [24]. Altogether, the advances presented by Crescitelli chart a path from descriptive EV isolation to clinically actionable diagnostics and next‐generation immunotherapies, firmly embedding the EV field within the translational cancer toolkit.

## Sessions

### EVs biogenesis and their function in signal transmission

Understanding the mechanisms regulating EV biogenesis and how the EV‐associated cargo mediates signaling activation is of critical importance to elucidate the processes of EV‐mediating intercellular communication. To investigate the molecular mechanisms behind EV uptake, Yáñez‐Mó et al. used a genome‐wide CRISPR screen in K562 leukemia cells, revealing the COMMANDER complex as a key player in endosomal trafficking and EV internalization. Additionally, Ludwig et al. explored the interaction between MSC‐EVs and galectins, which are carbohydrate‐binding proteins with immunomodulatory potential. It was observed that Galectin‐1 and Galectin‐3 are present in MSC‐EV preparations, suggesting a functional relationship. These findings suggest that Galectins interact with EVs *in vitro* and *ex vivo*, indicating a potential application for cell‐free therapies and immune modulation.

It has been reported that the HDAC6 inhibitor tubacin promotes EV release from cancer cells [29]. Thus, Médard et al. used a chemoproteomics approach to investigate the off‐target effects of tubacin. They identified MBLAC2 as a novel target, and its inhibition or silencing in HEK293 cells increased EV accumulation, suggesting a regulatory role in EV secretion. However, the molecular mechanisms by which MBLAC2 influences EV release remain to be elucidated. In line with ARF6 regulating the shedding of tumor‐derived EVs [30], Chabu et al. showed that KRAS/STK11 mutations in non‐small cell lung cancer activate ARF6, enhancing the release of immunosuppressive EVs that help tumors evade immune detection. Targeting ARF6 may improve immunotherapy outcomes. Notwithstanding, the molecular mechanisms by which KRAS and STK11 lead to ARF6 activation remain to be fully elucidated.

Németh et al. investigated how melanoma‐derived EVs influence tumor progression and resistance to therapy. Melanoma‐EVs were shown to enhance cell migration and proliferation and reduce the effectiveness of BRAF inhibitors, though not MEK inhibitors or combined BRAF/MEK therapy. Despite these findings, the specific EV‐associated mediators responsible for promoting tumor progression and resistance to BRAF inhibition remain to be identified. Furthermore, Nagy et al. examined how different neutrophil‐derived EVs affect monocytes and macrophages. EVs from apoptotic neutrophils enhanced monocyte viability and macrophage migration, while spontaneous PMN‐EVs showed anti‐inflammatory effects by reducing ROS, IL‐8, and CD11b expression. Both types promoted macrophage differentiation, suggesting a role in early inflammation regulation. Nevertheless, the specific cargo differences among neutrophil‐derived EVs subtypes and which components are responsible for the observed effects remain to be fully elucidated.

Rodrigues‐Junior et al. elucidated how transforming growth factor‐β (TGF‐β) signaling promotes tumor‐derived EV secretion [31]. Their findings revealed that TGF‐β activates the MEK–ERK1/2 pathway, leading to SREBP2 phosphorylation, inducing DHCR7 expression. This, in turn, enhances cholesterol biosynthesis, a critical factor in EV biogenesis [32]. TGF‐β stimulation affected the EV cargo, enriching MMP‐9 associated with EVs. Importantly, inhibition of MEK signaling or cholesterol synthesis reduced EV production and sensitized cancer cells to chemotherapy. These findings suggest that targeting the TGF‐β/MEK/cholesterol could limit EV‐mediated tumor progression, improving treatment response.

Notably, the poster of this session had 14 studies emphasizing the diverse roles of EVs in cell communication, disease processes, and therapeutic development. Topics included EVs in wound healing, embryo‐maternal signaling, cancer resistance, HIV‐related neuropathology, and Alzheimer's disease. Researchers examined EV molecular profiles, such as proteomics and lipidomics, and the effects of genetic alterations like tetraspanin or Rab27a deficiency.

### EVs in health and disease

This session highlighted the multifaceted role of EVs in disease development, intercellular communication, and the formation of pre‐metastatic niches. Additionally, it covered a broad spectrum of EVs connecting metabolic diseases, cancer therapy resistance, and immunomodulatory mechanisms. Since the cardiomyocyte‐derived EVs (CM‐EVs) function in myocardial regeneration remains underexplored [33], Borowski et al. expanded this discussion to post‐ischemic cardiac remodeling by investigating the role of CM‐EVs following myocardial infarction (MI). Using transgenic mouse models and a combination of proteomic analysis and *in vivo* imaging, they showed that CM‐EVs are enriched in mitochondrial proteins after MI and target splenic and pulmonary macrophages. Further assays confirmed that these EVs transfer Cre recombinase to recipient macrophages, as evidenced by GFP expression, indicating successful delivery of molecular cargo. Moreover, Kovácsházi et al. studied the impact of hypercholesterolemia (HC) on circulating and CM‐EVs in rats and human cells. HC altered EV profiles, reducing key phosphatidylcholines in circulation and causing proteomic changes in CM‐EVs. Despite increased EV secretion, monocyte activation was not observed, suggesting HC‐induced EVs may contribute to cardiac remodeling, offering potential for biomarker discovery in cardiovascular diseases.

In another context, Manon et al. revealed that EVs released from pancreatic islets under hyperglycemic conditions were enriched in *miR‐375‐3p* and trafficked to placental cells. In both human placental explants and pregnant mouse models, this EV‐mediated transfer of *miR‐375‐3p* was associated with increased placental and fetal growth. Proteomic analyses suggested that *miR‐375‐3p* affects placental development by altering key pathways involved in metabolism and growth, contributing to large‐for‐gestational‐age outcomes commonly observed in gestational diabetes mellitus pregnancies.

EVs are key messengers that enable communication between cancer and surrounding cells [34]. Expanding on the function of tumor‐derived EVs, Cooks et al. demonstrated that mutant p53‐containing EVs from pancreatic ductal adenocarcinoma cells promote organ‐specific metastasis by accumulating in the liver and lungs and remodeling the extracellular matrix. These EVs also alter the immune microenvironment by expanding granulocyte, monocyte, and myeloid‐derived suppressor cell populations, highlighting their dual role in metastasis promotion and immune modulation. Additionally, Costanzo et al. showed colorectal cancer (CRC)‐derived EVs assisting in the hepatic pre‐metastatic niche formation. Using a novel 3D‐spheroid model of healthy human hepatocytes, they demonstrated CRC‐EVs inducing epithelial‐mesenchymal transition (EMT) while reducing the expression of hepatocyte markers. These changes enhanced the invasiveness of CRC cells, underlining the active role of EVs in mediating pre‐metastatic niche formation. Furthermore, Loria et al. uncovered a mechanism in CRC metastasis involving EV‐mediated transfer of the long non‐coding RNA H19 and the splicing regulator RBFOX2 to hepatocytes. This dual RNA‐protein delivery induces alternative splicing changes linked to EMT, featuring EV cargo as a key regulator of gene expression related to pre‐metastatic niche formation.

Sagini et al. showed that exercise‐induced EVs (ex‐EVs) isolated from the plasma of trained human subjects or mice contained thioredoxin, which has been shown to slow breast cancer growth and enhance CD8^+^ T cell infiltration in mice. These findings suggest that ex‐EVs may mediate the anti‐tumor effects of exercise and could be developed as exercise‐mimetic therapies. Additionally, Kolatsi et al. showed that EVs isolated from paclitaxel (PTX)‐resistant triple‐negative breast cancer (TNBC) cells carried distinct sets of proteins linked to drug resistance and inflammation. Notably, proteins like ABCG2 and PRDX6 were enriched in PTX‐resistant cell‐derived EVs, while LPS stimulation induced a unique inflammatory protein signature. These findings indicate that TNBC EVs can act as both mediators and markers of chemoresistance and inflammatory stress, providing potential targets for therapeutic intervention.

Artemi et al. examined endothelial cell‐derived EVs in the context of chemotherapy‐induced cardiotoxicity, focusing on CRC treatment with 5‐fluorouracil. While 5‐fluorouracil reduced CRC cell viability, endothelial cells remained viable but exhibited altered EV secretion. EVs from treated endothelial cells showed reduced expression of CD9, CD63, and CD81, suggesting a disruption in EV biogenesis. These results propose endothelial cell‐derived EVs as biomarkers for chemotherapy‐induced toxicity, with potential for monitoring cardiovascular risk in cancer patients.

Izquierdo et al. uncovered that platelet‐derived EVs (PL‐EVs) influence immune and vascular responses in respiratory allergies. PL‐EVs from patients with varying allergy severities enhanced activated T cell proliferation, linked to disease severity, without affecting regulatory T cell numbers. Importantly, PL‐EVs disrupted endothelial barrier integrity, suggesting that PL‐EVs may intensify allergic inflammation by simultaneously activating immune cells and compromising vascular barriers.

Finally, the related poster session had innovative research highlighting the diverse roles of EVs in modulating inflammation, immune responses, tissue injury, or disease progression. These studies emphasized the therapeutic and diagnostic potential of EVs across a broad range of physiological contexts.

### EV‐based biomarkers

EV‐associated molecular cargo can provide biomarker‐guided therapeutic decisions, and this topic remains central in precision medicine, particularly when implemented through targeted therapy protocols [35]. This session brought diverse studies focused on the application of EVs as non‐invasive biomarkers for neurological, renal, and transplant‐related conditions. First, Vodušek et al. conducted a longitudinal study assessing urinary EVs (uEVs) and EV‐DNA as biomarkers for kidney allograft injury. Donor‐derived EV‐DNA levels effectively distinguished between healthy and injured grafts as early as 3 months post‐transplant. A multivariable model based on uEV features predicted injury with high specificity (96%), and its accuracy improved when combined with serum creatinine levels. Additionally, Vilardo et al. investigated EV signatures in amyotrophic lateral sclerosis (ALS), focusing on neuroprotective markers. GLAST^+^ and Proteoglycan‐4^+^ (PRG4^+^) EVs were elevated in ALS patients, with PRG4^+^ EVs particularly enriched in those with preserved cognitive function. Proteomic profiling revealed several proteins associated with inflammation and neurodegeneration, positioning these EVs as promising biomarkers for both disease progression and cognitive resilience in ALS. Anandan et al. presented pilot data showing that brain‐derived L1CAM^+^ EVs in multiple sclerosis patients are detectable in both cerebrospinal fluid and blood, with similar immunophenotypes. Treatment‐naïve patients had higher tetraspanin levels, while anti‐CD20 therapy shifted EV markers (CD8, CD19) toward healthy profiles. Detection of EBNA1 post‐treatment supports EVs as potential tools for monitoring immune status and treatment response. Furthermore, Bennett et al. highlighted technical challenges in isolating rare EVs via immunodepletion, showing that free proteins and buffer contaminants can distort western blot results. They stressed the need for careful marker validation and control of artifacts, especially in neuronal EV studies.

Stevanović et al. found that serum EVs from women with gestational diabetes mellitus had elevated levels of *miR‐146a‐5p* and *miR‐21‐5p*. Notably, *miR‐21‐5p* was correlated with inflammation, while *miR‐146a‐5p* was negatively correlated with anthropometric parameters of newborns, suggesting a link between EV microRNAs and fetal outcomes. Additionally, Almasri et al. demonstrated that the hypoxia marker CAIX is enriched in EVs from HER2^+^ breast cancer cells under low oxygen conditions, regardless of drug resistance. Its consistent presence in EVs suggests CAIX could serve as a non‐invasive sensor for tumor hypoxia, supporting EV application as diagnostic tools in cancer.

The poster session of EV‐based biomarkers field presented a diverse array of studies demonstrating the diagnostic, prognostic, and therapeutic monitoring potential of EVs across multiple disease contexts, including cancer, neurodegeneration, infection, and metabolic disorders. Together, these contributions underscore the growing relevance of EVs in precision medicine and liquid biopsy applications.

### EVs in interspecies communication

Understanding how EVs from diverse sources, such as helminths, insects, plants, rodents, and mammals interact with different species is crucial for uncovering conserved and unique mechanisms of interspecies communication, with implications for health, disease, and therapeutic development [36]. Thus, this session started with Alfandari et al. demonstrating that *Plasmodium falciparum*‐derived EVs are internalized by host immune cells via distinct mechanisms: T cells use a “capping pattern,” while monocytes rely on endocytosis. The EV uptake is influenced by the cells' biophysical properties, particularly cholesterol levels, suggesting that malaria parasites employ sophisticated intercellular communication strategies. Furthermore, Sabatke et al. investigated how *Giardia intestinalis*‐derived EVs are internalized by intestinal epithelial cells. They found that uptake is energy‐ and dose‐dependent, with EVs from host–parasite interactions being more efficiently internalized. Clathrin‐ and caveolin‐mediated endocytosis were identified as key pathways, suggesting potential therapeutic targets to disrupt parasite communication and reduce infection severity. Yet, in the context of malaria, Teruel et al. used bone marrow‐ and spleen‐on‐a‐chip models to study the role of malaria‐derived EVs on the parasite's cryptic infections. Their results showed that EVs from *P. vivax* and *P. falciparum* patients inhibited erythropoiesis and promoted parasite migration toward bone marrow when compared to healthy donors. These data provide novel insight into parasite niches and support the development of alternative malaria control strategies with a reduced need for animal use in human experimentation.

Schabussova et al. investigated the immunomodulatory effects of *Escherichia coli* O83‐derived EVs (EcO83‐EVs) in allergic airway inflammation. EcO83‐EVs activated cytokine production via NOD and TLR pathways in human and mouse immune cells. In a mouse allergy model, intranasal EV administration reduced eosinophilia and airway hyperreactivity, indicating a shift from Th2 to Th1 immunity. These findings highlight EcO83‐EVs as a promising therapeutic strategy for allergies and other inflammatory conditions. Lastly, Fenton et al. examined how EVs from cow's milk at different lactation stages affect human immune cells. They found that EVs influence ROS production and carry stage‐specific miRNA cargo, potentially modulating vascular inflammation and chronic inflammatory conditions.

The related poster session highlighted diverse roles of EVs in disease and interspecies communication. The highlights included bacterial outer membrane vesicles promoting lung cancer, lemon‐derived nanovesicles protecting the liver, and *Trichinella spiralis* EVs reducing allergic inflammation. Other studies revealed EVs' roles in microbial interactions, mental health biomarkers, and fungal‐bacterial communication, underscoring their broad biomedical and ecological significance.

### Novel EV preparation/analysis techniques

Studying methods for isolating or enriching EVs from various biological sources is essential to ensure the accuracy, reproducibility, and biological relevance of EV‐based research. These strategies are also critical for engineering EVs as delivery vehicles, where precise loading of therapeutic cargo and targeting specific cells can impact the efficacy and safety of EV‐based therapies [12]. This session contains presentations optimizing existing methodologies or developing novel promising approaches for EV research. Thus, Vanderpoorten et al. introduced NANOSPACERS, disposable nanofluidic capillaries that enable rapid size‐based classification of biological specimens using standard microscopy. The method covers a wide range, from motile bacteria to single molecules, and allows high‐throughput analysis of thousands of events within seconds. This cost‐effective approach could advance EV research in basic laboratory settings. Additionally, Shin et al. introduced a hybrid EV extraction method combining charge‐based ExoFilter technology, tangential flow filtration, and lipoprotein‐specific adsorption filters. This integrated approach yields ultra‐pure EVs with high recovery rates, scalable across volumes, and effectively removes lipoprotein contaminants. The system overcomes limitations of traditional methods by efficiently removing lipoprotein impurities, providing a powerful solution for producing high‐quality EVs for diverse applications.

Saari et al. developed a method for monitoring and purifying platelet‐derived EVs from donated blood using anion exchange chromatography combined with inline Raman spectroscopy. This technique enables real‐time molecular analysis during isolation, detecting impurities and process variations without labor‐intensive steps. The results align well with conventional methods, including nanoparticle tracking analysis (NTA), offering a streamlined approach to EV characterization. Filipović et al. applied a nanobody‐based immunoaffinity chromatography method to isolate EVs from multidrug‐resistant (MDR) cancer cells and their sensitive counterparts. Using VHHs‐GFP nanobodies immobilized on polymethacrylate, they captured EVs bearing small EV markers. Notably, MDR cells produced larger EVs than sensitive cells. The method is cost‐effective and adaptable for isolating EVs from various biological sources.

It remains challenging to identify novel sustainable and biologically safe sources of EVs for drug delivery [37]. In this context, Kocholata et al. proposed plant‐derived EVs as innovative drug delivery systems. The authors achieved the high yield and quality extraction of PDEVs from explants of *Nicotiana tabacum*, *Arabidopsis thaliana*, and other medicinal plants as model organisms. The PDEVs of tobacco contained the EV marker HSP70, RNS, and secondary metabolites. These PDEVs were successfully loaded with Cy5‐siRNA, YOYO‐pDNA, molybdenum cluster compounds, and doxorubicin, and applied to deliver EV‐associated cargo to a variety of plant and animal cells.

Parkkila et al. introduced waveguide scattering microscopy (WGSM) for label‐free, multiparametric profiling of single EVs, with simultaneous fluorescence readout. The technique requires minimal sample volume and enables analysis of thousands of EVs within40–80 min. WGSM distinguishes vesicles from non‐vesicular particles, measures size and refractive index, and quantifies biomolecules in the EV lumen. Its dual‐color staining capability allows tetraspanin marker detection, positioning WGSM as a promising tool for standardizing EV assays and advancing liquid biopsy applications. Another novel approach was presented by Yilmaz et al., introducing a microfluidic chip combined with a metamaterial sensor to enrich and detect EVs from cell cultures and urine samples. The method demonstrated high sensitivity and efficiency, validated by techniques like NTA, fNTA, immunoblotting, and scanning electron microscopy. It is user‐friendly, cost‐effective, and holds promise for EV‐based diagnostics in clinical settings. Finally, Botto et al. developed an optimized EV isolation protocol using small sample volumes (up to 15 mL), combining polymer‐based precipitation with size exclusion chromatography (PPT + ExoSpin). This method outperformed other tested workflows and is well‐suited for EV proteomics, offering potential for novel biomarker discovery.

The poster session had 20 innovative studies focusing on new methods for isolating brain‐ and plasma‐derived EVs, glycan profiling of tumor EVs, and EV‐based biosensors for detecting cancer and kidney disease. Several cost‐effective and scalable platforms—like 3D‐printed filters, microfluidic chips, and nanobody‐based assays—were introduced. Applications spanned diagnostics for Alzheimer's, HIV, cancer therapy monitoring, and gut health in ruminants, collectively advancing EV research and its clinical potential.

### EVs in therapy and regenerative medicine

The role of EVs in therapy and regenerative medicine is critical due to the unique EV ability to mediate intercellular communication, deliver therapeutic cargo, and modulate immune responses in a highly targeted and biocompatible manner [16]. This session started with Garofalo et al. introducing a novel treatment for malignant melanoma using an adenovirus‐based cancer vaccine delivered via EVs. This vaccine combines an oncolytic adenovirus with melanoma‐specific antigens. Imaging studies (both *in vivo* and *ex vivo*) showed that the vaccine effectively targeted primary and metastatic tumors, reduced tumor volume, and enhanced the immune response by increasing tumor‐infiltrating lymphocytes and activating cytotoxic T cells. Bozic et al. investigated the protective effects of MSC‐EVs in a mouse model of ischemia–reperfusion kidney injury (IRI). Eight‐week‐old C57BL/6 mice received intravenous MSC‐EVs after IRI. The treatment improved kidney function, reduced tissue damage, and lowered the expression of inflammatory markers (IL6, MCP‐1, IL1B, TNFa, RANTES). Their findings suggest that MSC‐EVs administration ameliorates IRI and mediates anti‐inflammatory effects in the kidney. Kostevšek et al. presented erythrocyte membrane‐based vesicles (EMVs) as siRNA carriers for safe and efficient gene silencing therapy. They loaded single or multiple siRNAs in EMVs, evaluating the purification strategy and storage techniques. Hence, they proposed a novel cost‐effective therapeutic nucleic acid carried by EVs that is prone to easy scaling‐up.

Tilotta et al. explored the anti‐senescence and anti‐inflammatory effects of citrus flavonoids Naringin and Hesperidin delivered via EVs in the context of intervertebral disc degeneration. They used immortalized bone marrow mesenchymal stem cells to load the flavonoids into EVs using three different methods, assessing loading capacity and encapsulation efficiency. The study highlights EVs as a promising bio‐nanomedicine with potential for targeted drug delivery, offering high chemical stability, low immunogenicity, and minimal toxicity.

The poster session presented studies exploring dendritic cell‐derived EVs for triple‐negative breast cancer, MSC‐EVs for arthritis and osteoarthritis, and algasomes as natural antimicrobials. Researchers also demonstrated EVs' potential in drug delivery, bone regeneration, and spine fusion using biomaterial scaffolds. Additional work examined EVs monitoring chronic graft‐versus‐host disease, cancer therapy, and inflammatory skin conditions. Advanced platforms like 3D cultures and biosensors were introduced for EV‐based therapy screening, highlighting EVs' growing role in personalized and cell‐free therapeutic strategies.

### Manufacturing of native and engineered EV products

Elucidating the manufacturing of native and engineered EVs products is crucial for consistency and scalability, purity and safety, functional optimization, regulatory compliance, and therapeutic versatility [38]. Recently, red blood cells‐derived EVs were used as a promising drug delivery system due to their high yield and bioavailability [39]. Thus, Ciferri et al. developed Nanoerythrosomes (NanoEs) for targeted cancer therapy using a two‐step method: surface functionalization with a fluorescent peptide via copper‐free click chemistry and drug loading with PTX through sonication. Functionalization was confirmed in ~ 50% of NanoEs, which showed higher uptake in EDB‐positive cells. Despite a low PTX loading efficiency (3%), the NanoEs effectively reduced cell viability, demonstrating therapeutic potential. Their study supports click chemistry and sonication as effective strategies for engineering NanoEs for targeted drug delivery.

Carreira et al. developed EVs loaded with gamma‐carboxylated Gla‐rich protein (cGRP), a vitamin K‐dependent protein with anti‐inflammatory and anti‐calcification properties, as a potential therapy for cardiovascular disease (CVD) [40]. They engineered two cell systems—baculovirus‐insect cells and human Expi293F cells—to produce cGRP‐loaded EVs. Vitamin K supplementation was essential for effective gamma‐carboxylation and enhanced cGRP loading. Both systems successfully produced therapeutic EVs, with Expi293F cells offering a scalable platform for EV‐based therapies targeting CVD.

Sonallya et al. investigated how host defense peptides (HDPs) interact with EVs to explore EV surface engineering. Using techniques such as flow cytometry, NTA, and TEM, they tested various HDPs, including LL37, Melittin, and Penetratin, detecting that LL37 and Lasioglossin removed EV surface proteins, Melittin disrupted membranes, while Octaarginine and Penetratin had minimal effects on EVs. Their findings offer a comprehensive biophysical analysis of HDP–EV interactions for therapeutic applications.

The poster session featured optimized EV isolation methods, such as chromatography, ultracentrifugation, and bioreactor systems, for improved scalability and purity. Highlights included engineering EVs with Klotho peptides to combat kidney fibrosis, functionalizing EV surfaces with hyaluronic acid derivatives, and using nanobody display for targeted cancer therapy. Additional work explored antimicrobial vesicles from *Lactiplantibacillus plantarum*, the effects of industrial processing on pomegranate‐derived nanovesicles, and the need for robust analytical pipelines to support scalable EV production.

## Conclusions

The 2nd MOVE Symposium showcased the dynamic landscape of EV research in Europe, highlighting advances in signaling, disease mechanisms, biomarkers, and therapeutic applications. Technological sessions introduced scalable methods for EV isolation and engineering with clinical potential. The meeting emphasized the importance of understanding EV heterogeneity, harmonizing methodologies, and preparing for clinical translation. A key strength of this meeting was its support for early‐career researchers, fostering mentorship and collaboration. The International Organizing Committee thanks all contributors, including speakers, presenters, sponsors, and the Serbian organizing team. Proceeds will support the MOVE Fellowship Program, promoting PhD mobility and cross‐border research. We look forward to MOVE‐2025 in Tartu, Estonia, from 7 to 10 October 2025, led by Alireza Fazeli, continuing MOVE's mission to drive collaborative EV research across Europe.

## Conflict of interest

The authors declare no conflict of interest.

## Author contributions

DMR‐J and MaK conceived and designed the project. DMR‐J, MiK, MEL, OJ, MY, and MaK wrote sections of the manuscript. DMR‐J, MiK, MEL, OJ, MY, and MaK reviewed and edited the manuscript.

## References

[1] Yu MSC , Edelbacher TV , Grätz C , Chiang DM , Reithmair M and Pfaffl MW (2024) Summary report of the 1st MOVE symposium in Málaga from 24‐27th October 2023 – Foster the European mobility for young scientists in extracellular vesicles research. Extracell Vesicles Circ Nucl Acids 5, 95–113. 39698417 10.20517/evcna.2024.09 PMC11648475

[2] Verweij FJ , Bebelman MP , George AE , Couty M , Bécot A , Palmulli R , Heiligenstein X , Sirés‐Campos J , Raposo G , Pegtel DM *et al*. (2018) ER membrane contact sites support endosomal small GTPase conversion for exosome secretion. J Cell Biol 221, e202112032. 10.1083/jcb.202112032 PMC9507465 36136097

[3] Verweij FJ , Bebelman MP , Jimenez CR , Garcia‐Vallejo JJ , Janssen H , Neefjes J , Knol JC , de Goeij‐de Haas R , Piersma SR , Baglio SR *et al*. (2018) Correction: quantifying exosome secretion from single cells reveals a modulatory role for GPCR signaling. J Cell Biol 217, 1157. 29362224 10.1083/JCB.20170320601192018c PMC5839778

[4] Mathieu M , Névo N , Jouve M , Valenzuela JI , Maurin M , Verweij FJ , Palmulli R , Lankar D , Dingli F , Loew D *et al*. (2021) Specificities of exosome versus small ectosome secretion revealed by live intracellular tracking of CD63 and CD9. Nat Commun 12, 4389. 34282141 10.1038/s41467-021-24384-2 PMC8289845

[5] Tertel T , Tomić S , Đokić J , Radojević D , Stevanović D , Ilić N , Giebel B and Kosanović M (2022) Serum‐derived extracellular vesicles: novel biomarkers reflecting the disease severity of COVID‐19 patients. J Extracell Vesicles 11, e12257. 35979935 10.1002/jev2.12257 PMC9451525

[6] Tertel T , Rebmann V , Bielefeld C , Haversath M , Jäger M , Wegner A , Busch A and Giebel B (2025) CD9+ and CD82+ extracellular vesicles in synovial fluid differentiate aseptic from septic endoprosthesis loosening. Extracell Vesicles Circ Nucl Acids 6, 336–349. 41142373 10.20517/evcna.2025.11 PMC12544082

[7] Eijndhoven MAJ , Scheepbouwer C , Aparicio‐Puerta E , Hackenberg M and Pegtel DM (2023) IsoSeek for unbiased and UMI‐informed sequencing of miRNAs from low input samples at single‐nucleotide resolution. STAR Protoc 4, 102645. 37858475 10.1016/j.xpro.2023.102645 PMC10594637

[8] Collot M , Ashokkumar P , Anton H , Boutant E , Faklaris O , Galli T , Mély Y , Danglot L and Klymchenko AS (2019) MemBright: a family of fluorescent membrane probes for advanced cellular imaging and neuroscience. Cell Chem Biol 26, 600–614.e7. 30745238 10.1016/j.chembiol.2019.01.009

[9] Hyenne V , Ghoroghi S , Collot M , Bons J , Follain G , Harlepp S , Mary B , Bauer J , Mercier L , Busnelli I *et al*. (2019) Studying the fate of tumor extracellular vesicles at high spatiotemporal resolution using the zebrafish embryo. Dev Cell 48, 554–572.e7. 30745140 10.1016/j.devcel.2019.01.014

[10] Wu AY‐T , Sung Y‐C , Chen Y‐J , Chou ST‐Y , Guo V , Chien JC‐Y , Ko JJS , Yang AL , Huang HC , Chuang JC *et al*. (2020) Multiresolution imaging using bioluminescence resonance energy transfer identifies distinct biodistribution profiles of extracellular vesicles and exomeres with redirected tropism. Adv Sci (Weinh) 7, 2001467. 33042758 10.1002/advs.202001467 PMC7539214

[11] Pachane BC , Carlson B , Queen SE , Selistre‐de‐Araujo HS and Witwer KH (2025) Exploring the adhesion properties of extracellular vesicles for functional assays. J Extracell Biol 4, e70042. 40292384 10.1002/jex2.70042 PMC12025881

[12] Welsh JA , Goberdhan DCI , O'Driscoll L , Buzas EI , Blenkiron C , Bussolati B , Cai H , di Vizio D , Driedonks TAP , Erdbrügger U *et al*. (2024) Minimal information for studies of extracellular vesicles (MISEV2023): from basic to advanced approaches. J Extracell Vesicles 13, e12404. 38326288 10.1002/jev2.12404 PMC10850029

[13] Zheng W , Rädler J , Sork H , Niu Z , Roudi S , Bost JP , Görgens A , Zhao Y , Mamand DR , Liang X *et al*. (2023) Identification of scaffold proteins for improved endogenous engineering of extracellular vesicles. Nat Commun 14, 4734. 37550290 10.1038/s41467-023-40453-0 PMC10406850

[14] Yan D , Haughton E , Liang X , El Andaloussi S , Wood M and Gupta D (2024) Engineered extracellular vesicles (EVs) for efficient delivery of crispr‐cas9 systems. Cytotherapy 26, S90.

[15] Liang X , Gupta D , Xie J , Wonterghem EV , Hoecke LV , Hean J , Van Wonterghem E , Van Hoecke L , Niu Z , Ghaeidamini M *et al*. (2025) Engineering of extracellular vesicles for efficient intracellular delivery of multimodal therapeutics including genome editors. Nat Commun 16, 4028. 40301355 10.1038/s41467-025-59377-y PMC12041237

[16] Giebel B , Kordelas L and Börger V (2017) Clinical potential of mesenchymal stem/stromal cell‐derived extracellular vesicles. Stem Cell Investig 4, 84. 10.21037/sci.2017.09.06 PMC5676188 29167805

[17] Marcilla A , Trelis M , Cortés A , Sotillo J , Cantalapiedra F , Minguez MT , Valero ML , Sánchez del Pino MM , Muñoz‐Antoli C , Toledo R *et al*. (2012) Extracellular vesicles from parasitic helminths contain specific excretory/secretory proteins and are internalized in intestinal host cells. PLoS One 7, e45974. 23029346 10.1371/journal.pone.0045974 PMC3454434

[18] Bernal D , Trelis M , Montaner S , Cantalapiedra F , Galiano A , Hackenberg M and Marcilla A (2014) Surface analysis of *Dicrocoelium dendriticum*. The molecular characterization of exosomes reveals the presence of miRNAs. J Proteomics 105, 232–241. 24561797 10.1016/j.jprot.2014.02.012

[19] Sánchez‐López CM , González‐Arce A , Soler C , Ramírez‐Toledo V , Trelis M , Bernal D and Marcilla A (2023) Extracellular vesicles from the trematodes *Fasciola hepatica* and *Dicrocoelium dendriticum* trigger different responses in human THP‐1 macrophages. J Extracell Vesicles 12, e12317. 37073796 10.1002/jev2.12317 PMC10114103

[20] Roig J , Saiz ML , Galiano A , Trelis M , Cantalapiedra F , Monteagudo C , Giner E , Giner RM , Recio MC , Bernal D *et al*. (2018) Extracellular vesicles from the helminth *Fasciola hepatica* prevent DSS‐induced acute ulcerative colitis in a T‐lymphocyte independent mode. Front Microbiol 9, 1036. 29875750 10.3389/fmicb.2018.01036 PMC5974114

[21] Trelis M , Galiano A , Bolado A , Toledo R , Marcilla A and Bernal D (2016) Subcutaneous injection of exosomes reduces symptom severity and mortality induced by *Echinostoma caproni* infection in BALB/c mice. Int J Parasitol 46, 799–808. 27590846 10.1016/j.ijpara.2016.07.003

[22] Sánchez‐López CM , Manzaneque‐López MC , Pérez‐Bermúdez P , Soler C and Marcilla A (2022) Characterization and bioactivity of extracellular vesicles isolated from pomegranate. Food Funct 13, 12870–12882. 36441623 10.1039/d2fo01806c

[23] Pérez‐Bermúdez P , Blesa J , Soriano JM and Marcilla A (2017) Extracellular vesicles in food: experimental evidence of their secretion in grape fruits. Eur J Pharm Sci 98, 40–50. 27664331 10.1016/j.ejps.2016.09.022

[24] Park K‐S , Svennerholm K , Crescitelli R , Lässer C , Gribonika I and Lötvall J (2021) Synthetic bacterial vesicles combined with tumour extracellular vesicles as cancer immunotherapy. J Extracell Vesicles 10, e12120. 34262675 10.1002/jev2.12120 PMC8254025

[25] Bagge RO , Berndtsson J , Urzì O , Lötvall J , Micaroni M and Crescitelli R (2023) Three‐dimensional reconstruction of interstitial extracellular vesicles in human liver as determined by electron tomography. J Extracell Vesicles 12, e12380. 38010190 10.1002/jev2.12380 PMC10680575

[26] Crescitelli R , Lässer C and Lötvall J (2021) Isolation and characterization of extracellular vesicle subpopulations from tissues. Nat Protoc 16, 1548–1580. 33495626 10.1038/s41596-020-00466-1

[27] Jang SC , Crescitelli R , Cvjetkovic A , Belgrano V , Bagge RO , Sundfeldt K , Ochiya T , Kalluri R and Lötvall J (2019) Mitochondrial protein enriched extracellular vesicles discovered in human melanoma tissues can be detected in patient plasma. J Extracell Vesicles 8, 1635420. 31497264 10.1080/20013078.2019.1635420 PMC6719261

[28] Crescitelli R , Filges S , Karimi N , Urzì O , Alonso‐Agudo T , Ståhlberg A , Lötvall J , Lässer C and Olofsson Bagge R (2022) Extracellular vesicle DNA from human melanoma tissues contains cancer‐specific mutations. Front Cell Dev Biol 10, 1028854. 36531960 10.3389/fcell.2022.1028854 PMC9751452

[29] Chao OS , Chang TC , Di Bella MA , Alessandro R , Anzanello F , Rappa G , Goodman OB and Lorico A (2017) The HDAC6 inhibitor tubacin induces release of CD133+ extracellular vesicles from cancer cells. J Cell Biochem 118, 4414–4424. 28452069 10.1002/jcb.26095

[30] Muralidharan‐Chari V , Clancy J , Plou C , Romao M , Chavrier P , Raposo G and D'Souza‐Schorey C (2009) ARF6‐regulated shedding of tumor cell‐derived plasma membrane microvesicles. Curr Biol 19, 1875–1885. 19896381 10.1016/j.cub.2009.09.059 PMC3150487

[31] Rodrigues‐Junior DM , Tsirigoti C , Psatha K , Kletsas D , Aivaliotis M , Heldin CH and Moustakas A (2025) TGF‐β induces cholesterol accumulation to regulate the secretion of tumor‐derived extracellular vesicles. J Exp Clin Cancer Res 44, 42. 39910665 10.1186/s13046-025-03291-0 PMC11800471

[32] van Niel G , D'Angelo G and Raposo G (2018) Shedding light on the cell biology of extracellular vesicles. Nat Rev Mol Cell Biol 19, 213–228. 29339798 10.1038/nrm.2017.125

[33] Gu J , Chen X , Luo Z , Li R , Xu Q , Liu M , Liu X , Liu Y , Jiang S , Zou M *et al*. (2024) Cardiomyocyte‐derived exosomes promote cardiomyocyte proliferation and neonatal heart regeneration. FASEB J 38, e70186. 39560071 10.1096/fj.202400737RR

[34] Lucotti S , Kenific CM , Zhang H and Lyden D (2022) Extracellular vesicles and particles impact the systemic landscape of cancer. EMBO J 41, e109288. 36052513 10.15252/embj.2021109288 PMC9475536

[35] Ragni E (2025) Extracellular vesicles: recent advances and perspectives. Front Biosci (Landmark Ed) 30, 36405. 40613286 10.31083/FBL36405

[36] Woith E , Fuhrmann G and Melzig MF (2019) Extracellular vesicles‐connecting kingdoms. Int J Mol Sci 20, 5695. 31739393 10.3390/ijms20225695 PMC6888613

[37] Fernández‐Rhodes M , Lorca C , Lisa J , Batalla I , Ramos‐Miguel A , Gallart‐Palau X and Serra A (2024) New origins of yeast, plant and bacterial‐derived extracellular vesicles to expand and advance compound delivery. Int J Mol Sci 25, 7151. 39000260 10.3390/ijms25137151 PMC11241179

[38] Paolini L , Monguió‐Tortajada M , Costa M , Antenucci F , Barilani M , Clos‐Sansalvador M , Andrade AC , Driedonks TAP , Giancaterino S , Kronstadt SM *et al*. (2022) Large‐scale production of extracellular vesicles: report on the “massivEVs” ISEV workshop. J Extracell Biol 1, e63. 38939213 10.1002/jex2.63 PMC11080784

[39] Drack A , Rai A and Greeninget DW (2023) Generation of red blood cell nanovesicles as a delivery tool. Methods Mol Biol 2628, 321–336. 36781795 10.1007/978-1-0716-2978-9_21

[40] Cavaco S , Viegas CSB , Rafael MS , Ramos A , Magalhães J , Blanco FJ , Vermeer C and Simes DC (2016) Gla‐rich protein is involved in the cross‐talk between calcification and inflammation in osteoarthritis. Cell Mol Life Sci 73, 1051–1065. 26337479 10.1007/s00018-015-2033-9 PMC11108449

